# Work-Related Musculoskeletal Disorders and Risk Factors among Chinese Medical Staff of Obstetrics and Gynecology

**DOI:** 10.3390/ijerph14060562

**Published:** 2017-05-26

**Authors:** Jingjing Wang, Ya Cui, Lihua He, Xiangrong Xu, Zhiwei Yuan, Xianning Jin, Zhimin Li

**Affiliations:** 1 Department of Occupational and Environmental Health, School of Public Health, Peking University Health Science Center, Beijing 100191, China; 1511210232@bjmu.edu.cn (J.W.); selina6887@163.com (X.X.); yuan90906236@126.com (Z.Y.); 18801238292@163.com (X.J.); 2 Shenzhen Prevention and Treatment Center for Occupational Diseases, Shenzhen 518020, China; cuiyacuiya@163.com

**Keywords:** obstetrics and gynecology staff, work-related musculoskeletal disorders, prevalence, risk factor, ergonomics

## Abstract

Medical staff in the department of obstetrics and gynecology are a group of professionals reportedly at high risk of work-related musculoskeletal disorders (WMSD), however, little is known about the current status of this problem in China. The aim of this study was to investigate prevalence and risk factors of work-related musculoskeletal disorders among this population in China. A self-developed questionnaire was distributed to 1017 obstetrics and gynecology practitioners to collect information on musculoskeletal symptoms and relevant factors. Prevalence and severity of work-related musculoskeletal disorders in different parts of the body were calculated and the relationship between personal and ergonomic factors and work-related musculoskeletal disorders was analyzed using Chi-square test and unconditional logistic regression models. The results indicated a high prevalence of 85.5% among the subjects, with the shoulder (*n* = 575, 62.0%), neck (*n* = 560, 60.3%) and lower back (*n* = 504, 54.3%) being the three most affected regions. Individual, postural, work-environmental as well as psychosocial factors were recognized to be associated with WMSDs in different body parts. Therefore, attention must be given to the problem of musculoskeletal disorders among Chinese obstetrics and gynecology staff. It is recommended to develop good life habits, improve work environment, adjust work organization as well as train on proper postures in their daily operation.

## 1. Introduction

**Figure 1:**
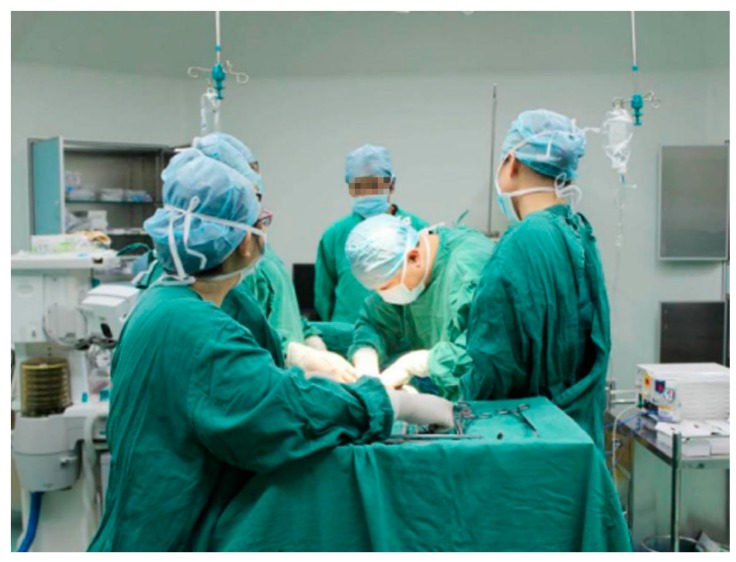
Obstetric or gynecological surgical procedures.

Obstetrics and gynecology practitioners are a group of working population affected by work-related musculoskeletal disorders (WMSDs). Their daily duties include but are not limited to practicing office-based procedures, carrying out surgeries and offering nursing care [1]. In these process, they have to meet the requirements of physical strength and professional knowledge [2], at the same time, they are exposed to fierce competition and tense physician-patient relationship [3]. Especially at the course of operations, they are required to transfer the heavy patients frequently and keep sustained periods of stooping, squatting, bending and constant trunk flexion (see Figure 1), which brings about pressure and posture load on neck, shoulder, and trunk etc. [4]. Consequently, the majority of them are exposed to poor ergonomic environment, which make them prone to musculoskeletal problems [1].

The U.S. Department of Labor defined work-related musculoskeletal disorders as injuries or disorders of the muscles, nerves, tendons, joints, cartilage, and spinal discs associated with exposure to risk factors in the workplace [5]. Data from the U.S. Bureau of Labor Statistics in 2015 showed that WMSDs were the most important parts of workers’ compensation, which accounted for at least one third of the labor time losses [6]. These diseases will not only affect the workers’ quality of life, but also impose a major economic burden to the society [7]. System review by Bruno et al. proposed that several biomechanical, psychosocial and individual factors contributed to the occurrence of WMSDs [8]. However, since the disorders are caused by a series of factors, it is difficult for researchers to fully elucidate the etiology.

Although previous studies have emphasized the serious problem of WMSDs and identified several work-related factors in obstetrics and gynecology [9], more information is needed. In particular, few study has focused on the prevalence of WMSDs and comprehensive ergonomic issues among Chinese obstetrics and gynecology staff. Therefore, the aim of this study was to investigate prevalence and severity of WMSDs, as well as the contribution of personal and ergonomic factors to the prevalence of WMSDs among this population in China, in order to provide them with valuable suggestions for intervention.

## 2. Methods

### 2.1. Instrumentation

A cross-sectional questionnaire survey was conducted. The questionnaire was designed by our research group and modified on the basis of the professional features of obstetrics and gynecology (see Supplementary Materials). The instrument has been done with reliability and validity test in the same population (total Cronbach’s alpha = 0.844, KMO (Kaiser-Meyer-Olkin) value = 0.872). Information collected in the questionnaire contains: (1) personal factors; (2) musculoskeletal symptoms; and (3) work-related factors.

As for personal factors, information concerning gender, age, vocation, length of employment, Body Mass Index (BMI), education, marital status, monthly income, smoking behavior and drinking behavior etc. was collected.

The second domain (Cronbach’s alpha = 0.895, KMO value = 0.616) captured information on musculoskeletal symptoms experienced in the past 7 days or 12 months in seven body regions: neck, shoulder, upper back, elbow, hand or wrist, lower back and knee, which were the most commonly-studied and vulnerable parts [1,10]. Furthermore, symptoms in the past 12 months were assessed by asking each subject to self-report pain frequency (1~7 days in the past year, 8~30 days in the past year, more than 30 days in the past year, almost every day) and pain intensity (score 0~10, with score 0 for no pain and score 10 for unbearable pain) in each body part. Information on total absenteeism time and the situation of changing job was also collected. The design of this domain was in accordance with the Standardized Nordic Musculoskeletal Questionnaire (NMQ) [11]. A case in the study refers to anyone who suffered from positive symptoms such as discomfort, numbness, pain or limitation of movement, that occurred in the musculoskeletal system at any time during the past 12 months, which lasted for at least 24 h and can’t get relief after rest [11,12,13].

Work-related factors involved information on postural, psychosocial and work-environmental factors. Postural factors (Cronbach’s alpha = 0.873, KMO value = 0.910) were constituted of five items on trunk, four items on neck, nine item on arm/wrist as well as three items on leg, which can be referred to Rapid Entire Body Assessment (REBA) [14]. Psychological factors (Cronbach’s alpha = 0.677, KMO value = 0.600) mainly focused on personal feelings, work organization and job control, which were partly drew from the full recommended version of the Karasek Job Content Questionnaire (JCQ) [15]. Finally, items on work environment (i.e., operating space, lumbar support, adjustable workbench, temperature and humidity) were collected.

### 2.2. Sampling and Data Collection

Medical staff who had worked in the department of obstetrics and gynecology for at least 1 year were recruited in our study. Those who had musculoskeletal injuries caused by sources other than workplace were excluded. There were 29 hospitals contacted that agreed to take part in the study. All of these hospitals were selected from hospitals of level II or above in different urban areas of Shenzhen. Thus, these 29 hospitals represented a convenience sample from the hospitals in Shenzhen, China. Between July 2015 and August 2015, the questionnaire with a cover letter explaining the purposes and procedure of the study was delivered to obstetrics and gynecology staff in these hospitals. Those who agreed to participate provided their signatures as informed consents. The questionnaire was completed under the guidance of trained investigators and went through strict quality control. Subjects who did not return filled questionnaire were contacted and encouraged to respond to the survey. Approval for all study procedures was obtained from the Ethics Committee of Henan Institute of Occupational Health (Approval codes: 2013003).

### 2.3. Data Analysis

Data was analyzed using SPSS 22.0 for Windows. Descriptive statistics were performed to reveal the response distribution for each item, especially the prevalence and severity of musculoskeletal symptoms in each anatomical site. Logistic regression analysis was carried out to evaluate the influence of individual and ergonomic factors on the occurrence of musculoskeletal symptoms in the past 12 months. Adjusted odd ratios (ORs) with 95% confidence intervals (95% CI) were obtained as measurement of association. For the initial selection of potential risk factors of musculoskeletal disorders, Chi square test was used with a significance level of *p* < 0.2. Subsequently, all independent variables that showed significant association were included in the multivariate logistic regression model. Age and gender were always included in each model regardless of its significance. The enter method was used for variable selection. These analyses were performed separately for different anatomical regions. The factor with OR > 1 was considered as a contributor toward WMSDs, whereas the factor with OR < 1 was considered as a protective factor. Finally, the potential for collinearity among risk factors was considered. The significance level of logistic regression was set to 0.05.

## 3. Results

### 3.1. Demographics Characteristics and Distribution of Ergonomic Factors

Of the 1017 questionnaires sent to subjects who were eligible to participate, there were 928 questionnaires returned and valid, yielding an response rate of 91.2%. Among the valid questionnaires, there were gynecologists (*n* = 288, 31.0%), obstetricians (*n* = 330, 35.6%), midwives (n = 310, 33.4%). The sex imbalance of respondents (68 male (7.3%) and 860 female (92.7%)) was comparable to that of the similar population in other studies [16]. A majority of the subjects were within the age group of 20~40 years (*n* = 675, 72.8%) and had a length of employment for more than 5 years (*n* = 672, 72.4%). Many of the subjects reported to work overtime (*n* = 852, 91.8%) and didn’t have regular (*n* = 724, 78.0%) or enough rest time (*n* = 679, 73.2%). About 78.4% (*n* = 728) of the participants often had to keep the same posture for long duration. Additional demographic characteristics and ergonomic factors were presented in Table 1.

### 3.2. WMSDs Characteristics

In total, there were 665 (71.7%) subjects who reported experiencing work-related musculoskeletal pain or discomfort in the past 7 days and 793 (85.5%) subjects reported WMSDs symptoms that occurred in at least one musculoskeletal region during the past 12 months. The most common symptoms appeared in the shoulder (*n* = 575, 62.0%), neck (*n* = 560, 60.3%) and lower back (*n* = 504, 54.3%). WMSDs severity was graded from no pain (score 0) to unbearable pain (score 10), with lower back, neck and shoulder being the most painful parts, whose mean scores were 5.3 ± 2.1, 5.3 ± 2.3 and 5.2 ± 2.2, respectively. Detailed data on the variables, such as cumulative duration of symptoms, absenteeism time and job change for the seven regions, were presented in Table 2.

### 3.3. Risk Factors Analysis

Results of risk factors with statistical significance in multivariate logistic regression models were presented in Table 3.

Several postural factors were found to be associated with musculoskeletal symptoms. “Uncomfortable posture” increased the prevalence of neck symptoms (OR = 1.497, 95% CI = 1.079, 2.077) and elbow symptoms (OR = 1.552, 95% CI = 1.040, 2.316) while “freely change posture” decreased lower back symptoms (OR = 0.729, 95% CI = 0.538, 0.989). Besides, “keeping the same posture for long time” (OR = 1.715, 95% CI = 1.182, 2.489) was related to increased prevalence of lower back pain. Elbow symptoms in particular were influenced by “arm placed on edges of angular objects” (OR = 1.542, 95% CI = 1.055, 2.256) and “tool size suitable for hand” (OR = 0.599, 95% CI = 0.391, 0.917) while hand/wrist symptoms were affected by “wrist flexion and extension frequently” (OR = 1.763, 95% CI = 1.102, 2.820) and “keeping shrugging for long period” (OR = 1.410, 95% CI = 1.041, 1.909).

Psychosocial factors involving personal feeling, work organization and job control were also recognized as risk factors in our study. Medical workers who perceived bad health status had increased odds of reporting shoulder pain (OR = 3.696, 95% CI = 1.834, 7.448), upper back pain (OR = 2.386, 95% CI = 1.247, 4.565) and hand/wrist pain (OR = 3.089, 95% CI = 1.632, 5.847) compared with those who reported good health. “Physical tiredness after work” increased the occurrence of lower back pain and hand/wrist pain and “mental tiredness after work” increased reported prevalence of knee symptoms. Employees who were able to “keep up with work pace” reported less neck symptoms (OR = 0.495, 95% CI = 0.361, 0.679), shoulder symptoms (OR = 0.650, 95% CI = 0.472, 0.895), lower back symptoms (OR = 0.610, 95% CI = 0.448, 0.832) and knee symptoms (OR = 0.476, 95% CI = 0.331, 0.684) than those who were not. In addition, neck symptoms in particular were related to “job stress” (OR = 1.494, 95% CI = 1.027, 2.172) and lower back symptoms in particular were affected by “enough rest time” (OR = 0.587, 95% CI = 0.396, 0.868).

Two work-environmental factors were recognized to be associated with musculoskeletal disorders, which were “feeling cold at work” (OR = 1.604, 95% CI = 1.065, 2.415) that correlated with neck symptoms and “adjustable workbench” (OR = 0.690, 95% CI = 0.497, 0.958) that related to upper back symptoms.

In spite of work-related factors, there were some personal factors found with statistical significance. We observed an increase with years of employment for the yearly prevalence of shoulder symptoms, mostly attributable to employees who had worked in the department of obstetrics and gynecology for 6~10 years (OR = 2.566, 95% CI = 1.492, 4.413). “Drinking behavior” showed correlation with elbow symptoms (OR = 1.706, 95% CI = 1.052, 2.765) and knee symptoms (OR = 2.303, 95% CI = 1.453, 3.649). Moreover, “monthly income” was found to be associated with neck symptoms and lower back symptoms while “marital status” was associated with back symptoms.

In the collinearity diagnostics, the Variance Inflation Factor (VIF) values of all variables were below 10, with tolerance values around 1 (shown in Table 4), indicating that there was no obvious collinearity problem among these potential risk factors.

### 3.4. Qualitative Feedback from the Participants

Our participants also provided important suggestions which deserve serious attention. Their comments mainly include: Firstly, it is necessary to raise benefits as well as reduce work intensity. Secondly, more attention should be paid to psychology, especially to creating harmonious relationship between doctors and patients. In addition, they complained about the unreasonable work organization, especially too many night shifts and training tasks, which made the already heavy work more unbearable. Finally, work environment was in need of improvement, since there were not enough operating tables and lounges.

## 4. Discussion

Medical staff are vulnerable to WMSDs [1]. Our findings revealed that WMSDs prevalence among obstetrics and gynecology staff was 85.5%, which was similar to the reported rate of 86.7% in research by Kim-Fine et al. [1], but rather high compared with many other vocations [17,18]. Therefore, WMSDs among obstetrics and gynecology practitioners should be a matter of concern. In our study, the most affected regions were shoulder, neck and lower back. It was slightly different from the ranking reported by Kierklo et al. [19], which were neck, lower back and hand among dentists. This may be attributed to the different occupational characteristics between these two departments, since in obstetric and gynecological surgeries, power requirement is more concentrated in the trunk rather than in the hand [20].

Our study also revealed that some personal factors as well as ergonomic factors were associated with prevalence of WMSDs even after mutual adjustment for each other, underscoring the multifactorial nature of WMSDs in this population.

Unfavorable postural factors were recognized to correlate with musculoskeletal symptoms in our study, which was inconsistent with Gangopadhyay, S. et al. [21]. According to some theories [22], when a person works in poor posture for long time, he will need to devote more strength to finishing the same intensity of task, which in turn increases the muscle loading and compressive stress on the vertebral disc and causes overwork injury over time. Besides, Armstrong et al. presented a conceptual model which may give us some hint on the pathogenesis of cumulative MSD [23]. Adverse posture may produce static load on the body. The static force exerting upon the musculoskeletal system may induce some physiological or biomechanical responses in the body, e.g., increased circulation, regional muscle fatigue, etc. Cumulative force requires continued or excessive responses, which might affect the reorganization or the regeneration procedure of the body tissue, causing structural tissue deformation. However, these speculations deserve further confirmation. Anyway, reducing posture load could be one of the most productive ways to alleviate WMSDs. Offering postural training as well as regular working posture evaluation and improvement were recommended in some studies [24].

Psychosocial factor is another crucial aspect of occupational hazards. The theory proposed by Carayon et al. that work organization and job stress may have comprehensive effects on the occurrence of WMSDs [25], was supported, in part, by the data from our study. According to some theoretical models describing the relationship between occupational factors and musculoskeletal problems like the dose-response model [26] or the biopsychosocial model [27], we assumed that psychosocial stressors at work may elicit some physiological responses, for example, increasing the individual’s muscle tension, and prolonged muscle tension can lead to the occurrence of musculoskeletal injury. Since one limitation of our study was that cross-sectional study may not be able to prove causality, we cannot assert that it was definitely poor mental health that caused WMSDs or it was WMSDs that affected mental health, or that maybe they contributed to each other. Therefore, more longitudinal studies to clarify this issue and verify our inference are required. However, in accordance with our study, adjusting work organization and paying attention to employees’ mental health may be advisable, especially developing a schedule which includes enough time for rest and an acceptable work pace, which are inconsistent with the subjective needs of medical staff as well [25].

Our study also demonstrated that “coldness” and “arm placed on edge of angular objects” increased the occurrence of WMSDs, while “adjustable workbench” and “tool size suitable for hands” decreased it. Based on these findings, we recommended that work environment should be improved and more and better equipment be offered to reduce WMSDs in the department of obstetrics and gynecology, especially providing some supportive devices, for example, adjustable seat and workbench, ergonomically styled surgical instruments, forearm support, etc. [28,29].

In addition, “length of employment” influenced the prevalence of neck pain, which was in agreement with Wang et al. [30]; this may be explained by the assumption that experienced medical workers are usually assigned to deal with more complex patients and surgery, which will be faced with heavier workload and a few of them develop musculoskeletal microtrauma from daily duties, which accumulates over time [31]. Other personal factors identified include “marital status” and “drinking behavior”. Thus, good living habits are also very important.

The limitations of this study should be acknowledged when interpreting the results. Firstly, there is no unified case definition of WMSDs worldwide, which may affect the comparability of results among studies. Secondly, the data presented here came from a convenience sample of 29 hospitals located in Shenzhen. Convenience sampling may result in estimates non-representative of workers in obstetrics and gynecology in general. Alternatively, healthy worker effect may exist in our study, as those who suffered from severe pain in musculoskeletal system may have already gone away from their post, thus were not included in our subjects. Finally, there may exist measurement error in self-assessment questionnaires. The aforementioned limitations indicated that our results should be interpreted with caution and further research on the mechanism and progress of WMSDs was warranted.

## 5. Conclusions

In conclusion, WMSDs and related factors among Chinese obstetrics and gynecology staff were surveyed in this study. Our results indicated a high prevalence rate of 85.5 % and that the shoulder, neck and lower back were the three most affected regions among this population. WMSDs are associated with individual, postural, work-environmental as well as psychosocial factors in obstetrics and gynecology. The findings can be used to guide prevention efforts for obstetrics and gynecology practitioners. Postural training, work organization adjustment, work environment improvement and healthy lifestyle were recommended for the prevention of WMSDs among them.

## Figures and Tables

**Table 1:** Demographics characteristics and distribution of ergonomic factors among subjects (*n* = 928).

Variables	Categories	N (%)
Gender	male	68 (7.3)
	female	860 (92.7)
Age (year)	20~30	294 (31.7)
	31~40	381 (41.1)
	41~50	179 (19.3)
	51~	74 (8.0)
Vocation	gynecologist	288 (31.0)
	obstetrician	330 (35.6)
	midwife	310 (33.4)
Length of employment (years)	1~5	256 (27.6)
	6~10	211 (22.7)
	11~15	155 (16.7)
	16~	306 (33.0)
BMI	~18.5	100 (10.8)
	18.5~24	670 (72.2)
	24~28	143 (15.4)
	28~	15 (1.6)
Education	senior high school and below	21 (2.3)
	junior college	136 (14.7)
	bachelor degree	585 (63.0)
	master degree or above	186 (20.0)
Marital status	unmarried	186 (20.0)
	married but separated	51 (5.5)
	married and living with spouse	691 (74.5)
Monthly income (RMB)	≤3000	48 (5.2)
	3001~5000	127 (13.7)
	5001~8000	342 (36.9)
	>8000	411 (44.3)
Smoking behavior	non-smoker	883 (95.1)
	past smoker	8 (0.9)
	current smoker	37 (4.0)
Drinking behavior	no	782 (84.3)
	yes	146 (15.7)
Weekly working hours	Mean (SD)	49.5 (13.2)
Shift work *	No	106 (11.4)
	Yes	822 (88.6)
Rest time	No	516 (55.6)
	Yes, not regular	353 (38.0)
	Yes, regular	59 (6.4)
Work overtime	No	76 (8.2)
	Yes	852 (91.8)
Physical tiredness after work	not at all	23 (2.5)
	a little bit tired	374 (40.3)
	tired	408 (44.0)
	can hardly bear	123 (13.3)
Mental tiredness after work	not at all	35 (3.8)
	a little bit tired	368 (39.7)
	tired	396 (42.7)
	can hardly bear	129 (13.9)
Perceived health status	good	113 (12.2)
	fine	673 (72.5)
	bad	115 (12.4)
	very bad	27 (2.9)
Maximum carrying weight (kg)	0~30	499 (53.8)
	30~60	258 (27.8)
	60~90	126 (13.6)
	90~	45 (4.8)
Enough operating space	No	255 (27.5)
	Yes	673 (72.5)
Lumbar support	No	376 (40.5)
	Yes	552 (59.5)
Adjustable workbench	No	620 (66.8)
	Yes	308 (33.2)
Freely change posture	No	490 (52.8)
	Yes	438 (47.2)
Keeping the same posture for long time	No	200 (21.6)
	Yes	728 (78.4)
Uncomfortable posture	No	396 (42.7)
	Yes	532 (57.3)
Coldness	No	689 (74.2)
	Yes	239 (25.8)
Feeling humid at work	No	778 (83.8)
	Yes	150 (16.2)
Enough rest time	No	679 (73.2)
	Yes	249 (26.8)
Rest regularly	No	724 (78.0)
	Yes	204 (22.0)
Control over work progress	No	684 (73.7)
	Yes	244 (26.3)
Job stress	No	226 (24.4)
	Yes	702 (75.6)
Keep up with work pace	No	454 (48.9)
	Yes	474 (51.1)

* “Shift work” refers to the workers taking turns on duty in different working hours according to schedule, which means that the employees sometimes need to work on public holidays or night shifts.

**Table 2:** Prevalence and severity of work-related musculoskeletal disorders (WMSDs) among Chinese obstetrics and gynecology staff.

Variables	N (% *)							
	Neck	Shoulder	Upper Back	Lower Back	Elbow	Hand/Wrist	Knee	Any Body Part
Occurrence								
in the past 7 days	485 (52.3)	474 (51.1)	267 (28.8)	430 (46.3)	144 (15.5)	293 (31.6)	218 (23.5)	665 (71.7)
in the past 12 months	560 (60.3)	575 (62.0)	330 (35.6)	504 (54.3)	187 (20.2)	374 (40.3)	261 (28.1)	793 (85.5)
Pain intensity								
Mean score (SD)	5.3 (2.3)	5.2 (2.2)	4.7 (2.1)	5.3 (2.1)	4.0 (2.4)	4.7 (2.2)	4.5 (2.2)	-
Cumulative duration of symptoms								
1~7 days	177 (19.1)	186 (20.0)	120 (12.9)	159 (17.1)	76 (8.2)	132 (14.2)	75 (8.1)	-
8~30 days	117 (12.6)	107 (11.5)	62 (6.7)	107 (11.5)	38 (4.1)	70 (7.5)	63 (6.8)	-
>30 days	179 (19.3)	164 (17.7)	86 (9.3)	140 (15.1)	39 (4.2)	97 (10.5)	72 (7.8)	-
Almost every day	70 (7.5)	77 (8.3)	38 (4.1)	69 (7.4)	15 (1.6)	41 (4.4)	30 (3.2)	-
No symptoms	368 (39.7)	353 (38.0)	598 (64.4)	424 (45.7)	741 (79.8)	554 (59.7)	667 (71.9)	-
Absenteeism time								
No absence	493 (53.1)	487 (52.5)	279 (30.1)	420 (45.3)	158 (17.0)	307 (33.1)	212 (22.8)	-
1~7 days	36 (3.9)	34 (3.7)	22 (2.4)	37 (4.0)	10 (1.1)	25 (2.7)	21 (2.3)	-
8~30 days	11 (1.2)	3 (0.3)	3 (0.3)	12 (1.3)	5 (0.5)	2 (0.2)	5 (0.5)	-
>30 days	5 (0.5)	12 (1.3)	6 (0.6)	8 (0.9)	4 (0.4)	8 (0.9)	6 (0.6)	-
No symptoms	368 (39.7)	353 (38.0)	598 (64.4)	424 (45.7)	741 (79.8)	554 (59.7)	667 (71.9)	-
Causing job change								
Yes	44 (4.7)	36 (3.9)	20 (2.2)	41 (4.4)	14 (1.5)	30 (3.2)	16 (1.7)	-
No	502 (54.1)	498 (53.7)	289 (31.1)	439 (47.3)	165 (17.8)	315 (33.9)	228 (24.6)	-
No symptoms	368 (39.7)	353 (38.0)	598 (64.4)	424 (45.7)	741 (79.8)	554 (59.7)	667 (71.9)	-

* Some percentages do not total 100 because of missing data.

**Table 3:** Risk factors analysis for WMSDs in different anatomical regions.

Regions	Factors	Categories	Significance	ORs	95% CI
Neck	Monthly income (RMB)	≤3000	0.018 *	1.000	-
		3001~5000	0.132	1.798	0.839 to 3.852
		5001~8000	0.686	0.865	0.429 to 1.745
		>8000	0.514	1.269	0.621 to 2.592
	Uncomfortable posture	binary ^a^	0.016 *	1.497	1.079 to 2.077
	Coldness	binary	0.024 *	1.604	1.065 to 2.415
	Job stress	binary	0.036 *	1.494	1.027 to 2.172
	Keep up with work pace	binary	0.000 **	0.495	0.361 to 0.679
Shoulder	Length of employment (years)	1~5	0.009 **	1.000	-
		6~10	0.001 **	2.566	1.492 to 4.413
		11~15	0.019 *	2.197	1.138 to 4.241
		16~	0.029 *	2.324	1.089 to 4.961
	Perceived health status	good	0.004 **	1.000	-
		fine	0.018 *	1.726	1.096 to 2.717
		bad	0.000 **	3.696	1.834 to 7.448
		very bad	0.281	1.781	0.624 to 5.088
	Keep up with work pace	binary	0.008 **	0.650	0.472 to 0.895
Upper back	Marital status	unmarried	0.034 *	1.000	-
		married but separated	0.241	0.738	0.444 to 1.226
		married and living with spouse	0.010 **	0.334	0.144 to 0.772
	Perceived health status	good	0.022 *	1.000	-
		fine	0.068	1.622	0.966 to 2.726
		bad	0.009 **	2.386	1.247 to 4.565
		very bad	0.012 *	3.585	1.326 to 9.697
	Adjustable workbench	binary	0.026 *	0.690	0.497 to 0.958
Lower back	Marital status	unmarried	0.025 *	1.000	-
		married but separated	0.706	0.912	0.566 to 1.470
		married and living with spouse	0.016 *	0.379	0.172 to 0.835
	Monthly income (RMB)	≤3000	0.003 **	1.000	-
		3001~5000	0.672	1.178	0.552 to 2.516
		5001~8000	0.787	1.104	0.539 to 2.260
		>8000	0.045 *	2.124	1.017 to 4.435
	Physical tiredness after work	not at all	0.021 *	1.000	-
		a little bit tired	0.108	2.571	0.812 to 8.142
		tired	0.048 *	3.356	1.013 to 11.117
		can hardly bear	0.005 **	6.729	1.765 to 25.662
	Freely change posture	binary	0.042 *	0.729	0.538 to 0.989
	Keeping the same posture for long time	binary	0.005 **	1.715	1.182 to 2.489
	Enough rest time	binary	0.008 **	0.587	0.396 to 0.868
	Keep up with work pace	binary	0.002 **	0.610	0.448 to 0.832
Elbow	Drinking behavior	binary	0.030 *	1.706	1.052 to 2.765
	Arm placed on edges of angular objects	binary	0.026 *	1.542	1.055 to 2.256
	Tool size suitable for hand	binary	0.018 *	0.599	0.391 to 0.917
	Uncomfortable posture	binary	0.032 *	1.552	1.040 to 2.316
Hand/wrist	Wrist flexion and extension frequently	binary	0.018 *	1.763	1.102 to 2.820
	Keeping shrugging for long period	binary	0.026 *	1.410	1.041 to 1.909
	Physical tiredness after work	not at all	0.014 *	1.000	-
		a little bit tired	0.357	1.956	0.470 to 8.146
		tTired	0.088	3.541	0.829 to 15.124
		can hardly bear	0.045 *	4.869	1.038 to 22.850
	Perceived health status	good	0.004 **	1.000	-
		fine	0.065	1.587	0.972 to 2.590
		bad	0.001 **	3.089	1.632 to 5.847
		very bad	0.641	1.259	0.477 to 3.321
Knee	Drinking behavior	binary	0.000 **	2.303	1.453 to 3.649
	Leg posture	sitting posture	0.028 *	1.000	
		keep both legs upright	0.007 **	2.237	1.240 to 4.036
		keep one leg upright with body weight on it	0.000 **	4.023	1.839 to 8.802
		squat with both legs	0.306	1.538	0.675 to 3.503
		squat with one leg	0.106	2.466	0.825 to 7.366
		kneeling position	0.067	3.444	0.917 to 12.934
		keep walking at work	0.014 *	2.109	1.164 to 3.819
	Mental tiredness after work	not at all	0.043 *	1.000	
		a little bit tired	0.935	0.956	0.325 to 2.808
		tired	0.758	1.192	0.390 to 3.641
		can hardly bear	0.125	2.619	0.765 to 8.964
	Keep up with work pace	binary	0.000 **	0.476	0.331 to 0.684

^a^ for binary variables, “yes” was marked as “1”, “no” was marked as “0”, OR is the prevalence odds ratio of “yes” group to “no” group. * *p* < 0.05; ** *p* < 0.01.

**Table 4:** Collinearity diagnostics among all variables.

Variables	Tolerance	VIF *
Gender	0.671	1.491
Age	0.260	3.846
Vocation	0.513	1.949
Length of employment	0.279	3.588
BMI	0.850	1.176
Education	0.690	1.448
Marital status	0.617	1.622
Monthly income	0.670	1.492
Smoking behavior	0.662	1.510
Drinking behavior	0.789	1.267
Trunk posture	0.715	1.399
Keep bending for long time	0.723	1.382
Turn round frequently	0.712	1.404
Keep trunk twisted for long time	0.615	1.625
Bend and turn at the same time frequently	0.571	1.750
Neck posture	0.747	1.338
Head remained low for long time	0.817	1.224
Keep your neck twisted for long time	0.623	1.604
Turn your head frequently	0.678	1.475
Wrist flexion and extension frequently	0.802	1.246
Twist your arm frequently	0.648	1.543
Have support device in your forearm	0.799	1.251
Keep your wrist twisted for long time	0.760	1.316
Arm placed on edges of angular objects	0.802	1.246
Keep shrugging for long period	0.835	1.198
Height of the arm	0.880	1.136
Tool size suitable for hand	0.857	1.167
Operating with both hands	0.885	1.129
Leg posture	0.836	1.196
Keep standing for long time	0.683	1.464
Keep your legs bent or twisted for long time	0.774	1.292
Weekly working hours	0.753	1.328
Shift work	0.860	1.163
Rest time	0.861	1.161
Work overtime	0.841	1.189
Physical tiredness after work	0.369	2.707
Mental tiredness after work	0.406	2.460
Perceived health status	0.777	1.287
Maximum carrying weight/kg	0.879	1.138
Enough operating space	0.811	1.233
Lumbar support	0.843	1.186
Adjustable workbench	0.855	1.170
Freely change posture	0.838	1.193
Keeping the same posture for long time	0.790	1.266
Uncomfortable posture	0.693	1.442
Coldness	0.704	1.421
Feeling humid at work	0.689	1.451
Enough rest time	0.592	1.688
Rest regularly	0.654	1.528
Control over work progress	0.727	1.375
Job stress	0.724	1.382
Keep up with work pace	0.773	1.294

* VIF is Variance Inflation Factor.

## Supplementary Materials

The following are available online at www.mdpi.com/1660-4601/14/6/562/s1, health status questionnaire.
