# Supplementary material for: Work-Related Musculoskeletal Disorders and Risk Factors among Chinese Medical Staff of Obstetrics and Gynecology
Source: Int J Environ Res Public Health. 2017 May 26;14(6):562. doi: 10.3390/ijerph14060562 (PMC5486248; doi:10.3390/ijerph14060562)
Supplement: Supplementary file 1 [file ijerph-14-00562-s001.pdf]

Supplementary file 1

Health status questionnaire

Thank you very much for reading and filling in this questionnaire. This questionnaire is to investigate current health status and work-related factors among obstetrics and gynecology staff.

Please answer each question in the questionnaire according to your personal situation. All the information you provide will be kept confidential and only be used for the purpose of this survey, please feel free to answer it!

If you agree to participate in this investigation, please sign your name. Thanks again for your cooperation!

Signature:

General information

1. Gender: ①male; ②female.
2. Age: ______.
3. Vocation: ①gynecologist; ②obstetrician; ③midwife.
4. Length of employment in the department of obstetrics and gynecology:____years.
5. Height:______cm; weight:______kg.
6. Education: ①senior high school and below; ②junior college; ③bachelor degree; ④master degree or above.
7. Marital status: ①unmarried; ②married but separated; ③married and living with spouse.
8. Monthly income: ①≤3000; ②3001～5000; ③5001～8000; ④＞8000.
9. Smoking behavior: ①non-smoker; ②past smoker; ③current smoker.
10. Drinking behavior: ①no; ②yes.

Musculoskeletal symptoms

1. Please answer the questions on musculoskeletal symptoms in the table below.

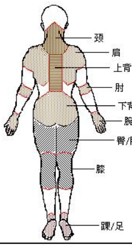	In the past 7 days, have you ever had musculoskeletal symptoms (discomfort, numbness, pain or limitation of movement) in the following regions (lasted for at least 24 hours)?	In the past 12 months, have you ever had musculoskeletal symptoms (discomfort, numbness, pain or limitation of movement) in the following regions (lasted for at least 24 hours) ?
Neck		
Shoulder		
Upper back		
Lower back		
Elbow		
Hand/wrist		
Knee		

2. If you have experienced musculoskeletal symptoms in the past 12 months, please answer the following questions, otherwise, skip the table.

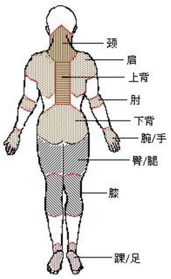	Pain intensity 0~10<br>0 for no pain;<br>10 for unbearable pain.	Cumulative duration of symptoms in the past 12 months<br>①1~7 days;<br>②8～30 days;<br>③>30 days;<br>④almost everyday.	Absenteeism time in the past 12 months<br>①no absence;<br>②1～7 days;<br>③8～30 days;<br>④>30 days.	Have you ever changed jobs or tasks because of musculoskeletal symptoms?<br>①no;<br>②yes.	Have you ever been injured in the accident?<br>①no;<br>②yes.
Neck					
Shoulder					
Upper back					
Lower back					
Elbow					
Hand/wrist					
Knee					

Working posture

Trunk

1. Which of the following best describes your trunk posture at work?
   ①flexion 0~20°　　②flexion 20~60°　　③flexion over 60°

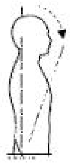
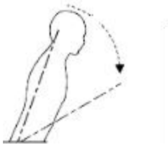
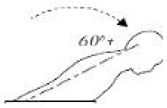

2. Do you keep bending for long time at work? ①yes; ②no.
3. Do you turn round frequently at work ? ①yes; ②no.
4. Do you keep trunk twisted for long time at work? ①yes; ②no.
5. Do you bend and turn at the same time frequently? ①yes; ②no.

Neck

1. Which of the following best describes your neck posture at work?
   ①flexion <10°　　②flexion 10~20°　　③flexion >20°　　④extension

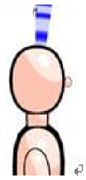
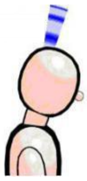
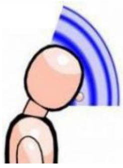
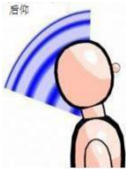

2. Does your head remain low for long time at work? ①yes; ②no.
3. Do you keep your neck twisted for long time at work ? ①yes; ②no.
4. Do you turn your head frequently? ①yes; ②no.

Arm and wrist

1. Do you often flex and extend your wrist frequently? ①yes; ②no.
2. Do you often twist you arm? ①yes; ②no.

3. Do you have support device in your forearm at work? ①yes; ②no.
4. Do you keep your wrist twisted for long time? ①yes; ②no.
5. Do you place your arm on edges of angular objects? ①yes; ②no.
6. Do you keep shrugging for long period ? ①yes; ②no.
7. What is the usual height of the arm?
   ①below shoulder ; ②above shoulder .
8. Is the tool size suitable for hand ? ①yes; ②no.
9. Do you usually operate with both hands? ①yes; ②no.

Leg

1. Which of the following best describes your leg posture at work?
   ①sitting posture ; ②keep both legs upright;
   ③keep one leg upright with body weight on it; ④squat with both legs;
   ⑤squat with one leg ; ⑥kneeling position;
   ⑦keep walking at work.
2. Do you keep standing for long time? ①yes; ②no.
3. Do you keep your legs bent or twisted for long time? ①yes; ②no.

Psychosocial factors and working environment

1. How long do you work every day?_______How many days do you work every week?_______.
2. Do you have shift work? ①yes; ②no.
3. Do you have rest time in your working hours?
   ①no; ②yes, not regular; ③yes,regular.
4. Do you often work overtime? ①yes; ②no.
5. How do you feel physical tiredness after work?
   ①not at all; ②a little bit tired; ③tired; ④can hardly bear.
6. How do you feel mental tiredness after work?
   ①not at all; ②a little bit tired; ③tired; ④can hardly bear.
7. How do you feel about your health status: ①good; ②fine; ③bad; ④very bad.
8. What is the maximum carrying weight at work?________kg
9. Do you think there is enough operating space at work? ①yes; ②no.
10. Do you have lumbar support on your seat ? ①yes; ②no.
11. Do you have adjustable workbench ? ①yes; ②no.
12. Can you change your posture freely at work ? ①yes; ②no.
13. Do you keep the same posture for most of the working hours ? ①yes; ②no.
14. Do you feel your working postures uncomfortable? ①yes; ②no.
15. Do you feel cold at work? ①yes; ②no.
16. Do you feel humid at work ? ①yes; ②no.
17. Do you feel there is enough rest time ? ①yes; ②no.
18. Is it possible to rest regularly ? ①yes; ②no.
19. Do you have control over your work progress ? ①yes; ②no.
20. Do you feel stressed at work? ①yes; ②no.
21. Do you feel hard to keep up with work pace ? ①yes; ②no.
22. Based on the current situation, do you have any suggestions for improvement?
